# From oncogenesis to prognosis: the roles of the immunoproteasome in cancer

**DOI:** 10.3389/fimmu.2025.1603816

**Published:** 2025-07-08

**Authors:** Delphine Béland, Mélissa Viens, Emma Mary Kalin, Marie-Claude Bourgeois-Daigneault

**Affiliations:** ^1^ Cancer Axis and Institut du cancer de Montréal, Centre Hospitalier de l’Université de Montréal (CHUM) Research Centre, Montreal, QC, Canada; ^2^ Immunopathology Axis, Centre Hospitalier de l’Université de Montréal (CHUM) Research Centre, Montreal, QC, Canada; ^3^ Department of Microbiology, Infectious Diseases and Immunology, Faculty of Medicine, University of Montreal, Montreal, QC, Canada

**Keywords:** immunoproteasome, cancer, oncogenesis, anti-tumor immunity, clinical outcome

## Abstract

The proteasome (prosome, macropain) is a key cellular organelle responsible primarily for protein homeostasis, by degrading damaged or misfolded proteins. Proteasome-processed protein fragments can then be further trimmed and funneled to the major histocompatibility complex class I (MHC-I) antigen presentation pathway for cell surface display and immune recognition. Various types of proteasomes can be found in mammalian cells with different expression patterns and cleavage abilities. As such, the immunoproteasome (ImP) preferentially cleaves proteins to yield MHC-I-compatible fragments. It is constitutively expressed by some immune cells and can be induced by pro-inflammatory signals. Interestingly, it was also found to be expressed in multiple types of cancers and proteasome activity can be modulated by some cancer therapies. A better understanding of its impact on cancer progression, prognosis and treatment response is therefore needed to guide treatment decisions. In this review, we focus on the multiple roles of the ImP in cancer, including its interplay with the immune system, as well as its impact on patient outcomes.

## 1 Introduction

**Figure 1:**
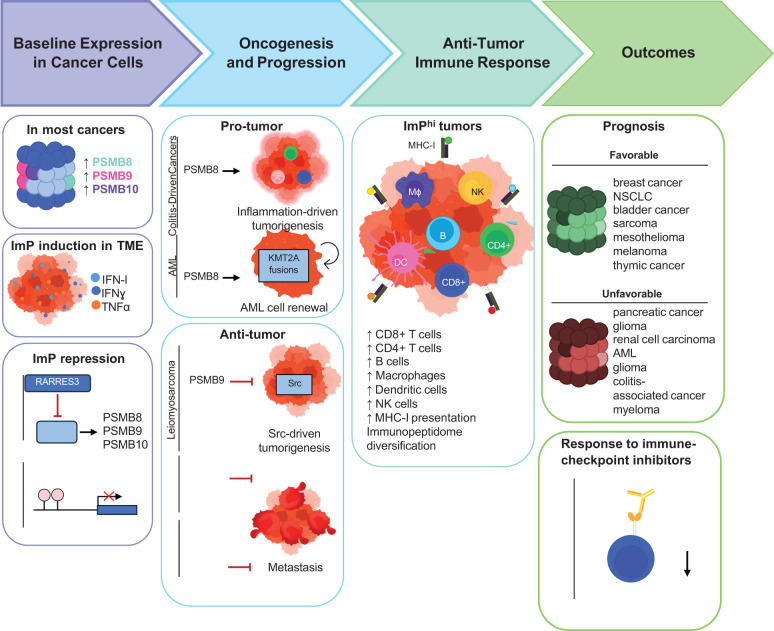
Schematic representation of the roles of the ImP across different stages of cancer progression. Most cancers exhibit increased ImP expression compared to healthy tissues while some others repress ImP expression. The known mechanisms by which cancers regulate ImP expression are depicted. The effects of the ImP in oncogenesis, anti-tumor immunity and outcomes are also outlined.

The constitutive 26S proteasome (CP) is a large intracellular proteolytic complex that is primarily known for its key role in protein homeostasis (1). Protein degradation by the CP allows for the recycling of misfolded and damaged proteins and also modulates various cellular functions by degrading key pathway regulators, as well as other components (2–4). Importantly, the CP also contributes to antigen presentation by major histocompatibility class I (MHC-I) molecules. Indeed, it cleaves proteins into peptides that are then fed into the pathway for loading onto MHC-I molecules, cell surface display and presentation to immune cells (5). Other types of proteasomes are also found in mammals. As such, the thymoproteasome, the spermatoproteasome and the ImP are expressed in different contexts. While the spermatoproteasome is present in the testes during spermatogenesis, and the thymoproteasome is expressed in cortical thymic cells, the ImP is expressed by some immune cells and also inducible by most cell types in inflammatory conditions (6). The ImP is also found in many cell types in the tumor microenvironment (TME), where it was described to impact disease outcomes (7). In this review, we discuss the various roles of the ImP through cancer progression (**Figure 1**).

## 2 ImP structure

All proteasomes contain a barrel-shaped 20S core particle, which is composed of four heptameric rings. The two outer rings are composed of proteasome 20S subunit alpha 1 to 7, which are encoded by the proteasome 20S subunit alpha (*PSMA*) genes. The alpha rings are conserved across all proteasome subtypes and guard access to the inner catalytic chamber, where cleavage takes place (8). The two inner rings are composed of proteasome subunit beta type 1-7 (PSMB1-7), which have either structural (PSMB1-4) or catalytic roles. More specifically, PSMB5, 6 and 7, bear chymotrypsin-, caspase- and trypsin-like cleavage abilities, respectively (9). While chymotrypsin-like activity cleaves proteins after residues with hydrophobic side chains such as tyrosines, leucines, isoleucines and phenylalanines, caspase-like cleavage cuts after acidic residues like asparagines and glutamates and trypsin-like cleavages takes place after basic residues like arginines and lysines (10, 11). Notably, chymotrypsin-like cleavage yields protein fragments with optimal residues for anchoring to the MHC-I peptide-binding groove. For the ImP, the catalytic subunits are PSMB8 (also called low molecular mass peptide (LMP) 7 or ImP subunit β5 (PSβ5i)), PSMB9 (LMP2 or PSβ1i) and PSMB10 (multi-catalytic endopeptidase complex subunit 1 or PSβ2i) (12). While PSMB8 and 10 retain the activities of their CP homologs, PSMB9 provides additional chymotrypsin-like cleavage to the ImP instead of trypsin-like activity (**Table 1**) (12). As a result, the CP and ImP generate different repertoires of peptides, which can then be loaded onto MHC-I molecules (13). This surface display of the intracellular protein content allows for immune cells to identify and eliminate cells that are infected or mutated. This is especially important in the context of cancer where immunoediting allows for the elimination of malignant cells (14). As described below, the ImP was found to affect cancer progression and treatment responses in a variety of cancer models (15, 16). It is expressed by different cell types and its aberrant regulation can affect cellular function that can be either beneficial or detrimental to the disease, depending on the context.

**Table 1:** Catalytic activities of CP and ImP subunits.

CP subunit	ImP subunit	Cleavage type	Cleavage after amino acids
PSMB5	PSMB8	Chymotrypsin-like	Hydrophobic (tyrosine, leucine, isoleucine, phenylalanine)
PSMB6		Caspase-like	Acidic (asparagine, glutamate)
	PSMB9	Chymotrypsin-like	Hydrophobic (tyrosine, leucine, isoleucine, phenylalanine)
PSMB7	PSMB10	Trypsin-like	Basic (arginine, lysine)

## 3 ImP regulation

**Figure 2:**
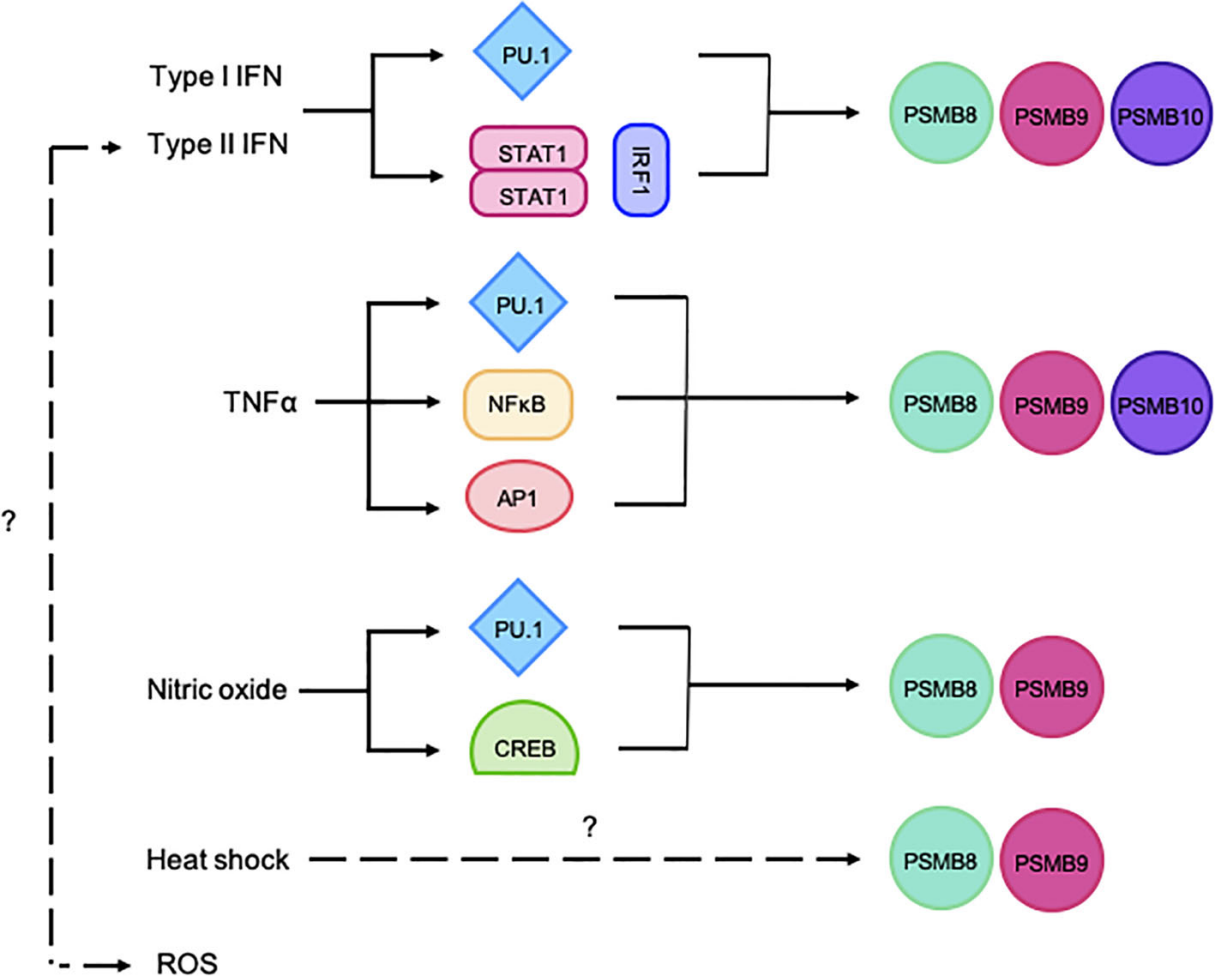
Schematic representation of the regulation of ImP subunit expression. The ImP subunits can be induced via PU.1, STAT1/IRF1, NF-κB, AP1 or CREB. The figure illustrates various stimuli that trigger the expression of PSMB8, 9 and/or 10.

Hematopoietic cells tend to have a proteasome pool that is skewed towards ImPs. Notably, the ImP is constitutively expressed by professional antigen-presenting cells such as dendritic cells, B cells and macrophages, therefore supporting its importance for antigen presentation (17). These cells, as well as T cells, NK cells, granulocytes, and monocytes, almost exclusively express the ImP (18). For non-immune cells, ImP expression can be induced by pro-inflammatory cytokines (19), with type-II interferon (IFNγ) being the most potent inducer (20). Type-I IFNs, as well as tumor necrosis factor α (TNFα) (20, 21) and various stress signals such as a heat shock, reactive oxygen species, nitric oxide and bacterial lipopolysaccharide can also trigger its expression (**Figure 2**) (22–25).

*PSMB8* and *9* genes are closely encoded in the MHC-II locus on chromosome 6 in humans or 17 in mice and are therefore co-regulated (12). Their promoters contains multiple sequence elements that are recognized by signal transduction and activator of transcription 1 (STAT1), as well as IFN regulatory factor 1 (IRF1), thus conferring IFN-responsiveness (26). Alternatively, *PSMB8* and *9* transcription can also be induced by nuclear factor kappa-light-chain enhancer of activated B cells (NF-κB), cyclic adenosine monophosphate responsive element binding protein (CREB), zinc finger protein 268 (ZNF268), specificity protein 1 (SP1), as well as activator protein 1 (AP1/2), therefore allowing for their IFN-independent regulation (27, 28). *PSMB10* is located on a different chromosome (8 in humans and 16 in mice), but can also be induced by STAT1, IRF1, NF-κB, SP1 and AP1/2 (12). Finally, IFNs, TNFα and nitric oxide can also induce the expression of all three ImP subunits via purine-rich box binding protein 1 (PU.1) (29–32) (**Figure 2**). When expressed, PSMB8, 9 and 10 are preferentially incorporated into the proteasome complex. Importantly, the subunits contain N-terminal pro-peptides that block their catalytic sites, which prevents aberrant protein cleavage (33). Once the complex is fully assembled, the pro-peptides are cleaved, and the ImP is catalytically active (3). Functional ImPs accomplish various functions that, in addition to shaping immune responses, also affect cancer onset, progression and treatment responses.

## 4 ImP functions within the tumor niche

The TME is a complex network that includes several cell types, stroma, as well as extracellular matrix. Given that the ImP is a cellular component that is not secreted, it is neither found in the stroma nor the extracellular matrix. Instead, it can be found in the cells of the TME, which include immune cells, endothelial cells, cancer-associated fibroblasts (CAFs) and tumor cells themselves (7, 34–36).

Immune cells infiltrate tumor tissues and act as critical modulators of immune surveillance (7, 37, 38). To avoid immune recognition, many cancers have therefore evolved defects in various components of the antigen presentation machinery. For example, non-small cell lung cancer (NSCLC), as well as pancreatic and esophagus squamous cell carcinomas downregulate ImP expression (39–41). Importantly, many studies are now describing the ImP as a biomarker of tumor immunogenicity (7, 42–44). For instance, in lower-grade glioma tumors, PSMB9 transcript levels were shown to correlate with increased gene signatures for CD8 and CD4 T cells, macrophages, as well as B cells (45). Similarly, Kumar et al. conducted a pan-cancer analysis of PSMB8, 9 and 10 expression and immune infiltration and found that PSMB8, 9 and 10 co-expression was linked to increased CD8 T cell, natural killer cell and activated dendritic cell gene signatures in most cancer types. Finally, it has been shown in myeloma, melanoma and clear cell renal cell carcinoma that activating the ImP increased the presentation of tumor-associated antigens, which resulted in enhanced anti-tumor activity (42, 46, 47).

Although the role of the ImP in antigen presentation is recognized as its main function, it also impacts immune cells in various other ways. For dendritic cells, ImP expression was shown to shape their cellular transcriptome. Indeed, PSMB8- and 10-deficient cells exhibited decreased activation of IRFs, STATs and NF-κB, which in turn prevented the expression of multiple-pro-inflammatory genes (48), which may impair the recruitment, activation and polarization of adaptive immune cells with anti-tumor activity within the TME (49).

For macrophages, high PSMB9 expression within the tumor was linked to increased phagocytosis (50), which could suggest enhanced tumor clearance and subsequent presentation of cancer antigens. For CD8 T cells, PSMB8, 9 and 10 co-expression was linked to increased activation and intra-tumor infiltration (49). For CD4 T cells, PSMB8 was shown to be critical for their activation by allowing for optimal MHC-II presentation (51). Furthermore, an ImP inhibitor was shown to favor the differentiation of T cells into regulatory T cells rather that Th1 or Th17 cells (52, 53). PSMB8 was also found to contribute to cell activation and protein homeostasis in both CD4 T cells and B cells (52).

For natural killer (NK) cells, which recognize missing self, their cytotoxic activity is increased against ImP-deficient cells because they express lower levels of surface MHC-I molecules. While this enhanced NK cell activity was studied in the context of influenza virus infection (54), the opposite was found in the context of cancer for which a single cell transcriptomics study revealed elevated activity of NK cells in melanoma tumors expressing high PSMB9 (50). Further work will be needed to better understand the importance of the ImP for NK cells in the context of cancer.

While ImP-mediated enhanced immune cell infiltration can be beneficial for immunogenic cancers, it can also be deleterious for inflammatory cancers such as colitis-induced colorectal carcinoma for which the recruitment and pro-tumoral polarization of immune cells sustain inflammation (55). For instance, PSMB8 was shown to increase the expression of chemokines that attract neutrophils, such as C-X-C motif ligand 1, 2 and 3, as well as vascular cell adhesion molecule 1, which was shown to support inflammation-driven tumorigenesis (55). The impact of the ImP on immune infiltration and cancer immunogenicity can also influence the response to therapy, as this infiltration was identified as important for the efficacy of immune checkpoint inhibitors (56, 57). For instance, NSCLC, melanoma, as well as muscle-invasive bladder, breast and thymic cancers expressing high levels of PSMB8, 9 and 10 were shown to respond better to the treatment (7, 58). PSMB9 was found to be more important in bladder cancer (59) and PSMB8 and 9 in melanoma (42).

Additional functions of the ImP in immune cells are summarized in **Table 2**. While some of these functions were described in non-cancer contexts and remain to be fully understood, they are likely to also take place within the TME (60, 61). Of note, while PSMB8 and 9 were the first ImP subunits to be discovered (62), PSMB10 was identified later (63) and remains overall less characterized. As such, additional functions in various immune cells will likely be uncovered in the future.

**Table 2:** Impact of ImP subunits on immune cells found within the TME.

Immune cell type	ImP subunit	Impact
CD8 T cells	PSMB8 PSMB9 PSMB10	CD8 T cell activation through enhanced MHC-I presentation (34, 46, 92)
CD4 T cells	PSMB8	CD4 T cell activation through enhanced MHC-II antigen presentation (51) Favors differentiation into Th1 or Th17 over Tregs (52, 53) Maintenance of protein homeostasis (52)
B cells	PSMB8 PSMB9	Altered antigen processing (97)
	PSMB8	B cell activation (52) Maintenance of protein homeostasis (52)
Dendritic cells	PSMB8 PSMB10	Increased IRF, STAT and NF-κB activity yields an altered transcriptome (48)
	PSMB8 PSMB9	Altered antigen presentation (96)
Macrophages	PSMB9	Increased phagocytosis by tumor-associated macrophages in melanoma (50)
NK cells	PSMB8 PSMB10	Facilitates recognition of infected cells by NK cells (54)

For fibroblasts, the ImP can be induced by IFNγ and interestingly, this induction is lost in the context of senescence (64). Since senescence is often observed in cancer in response to treatments, CAFs might have defects that prevent ImP induction. Also, PSMB8-deficient fibroblasts of the skin have altered responses to oxidative stress, supporting an important role of the ImP in maintaining protein homeostasis (25). While we could not find any study specifically delineating the functions of the ImP in CAFs, its impacts in other types of fibroblasts might also translate in a cancer setting.

Similarly to CAFs, the impact of the ImP on tumor endothelial cells is understudied. In endothelial cells, the ImP can once again be induced by IFNγ (65) and ImP expression by these cells was shown to contribute to CD8 T cell activation and tissue infiltration in the context of hypertension (66). Once again, further studies are needed determine if this also occurs in tumor endothelial cells. As for Imp functions in tumor cells themselves, these are detailed in section 3.

## 5 ImP modulation in cancer

One hallmark of cancer is genetic instability (67). As such, cancer cells accumulate mutations, some of which perturb cellular processes and aberrant gene expression is often observed. Interestingly, the basal expression of PSMB8, 9 and 10 is often augmented in bladder, breast, head and neck, and subtypes of renal cancers compared to healthy tissues (45, 68). PSMB8 and 9 are also overexpressed in lung, pancreatic, stomach, colon, prostate, thyroid, liver, uterine, cervical, testicular, ovarian and rectal cancers, as well as in diffuse large B cell lymphoma, acute myeloid leukemia, glioma, glioblastoma, cholangioma and melanoma (45, 68–70). ImP expression can also be heterogenous within a tumor. For instance, a study found high expression of PSMB8 in only 20% of NSCLC cells (39). ImP expression can also vary across cancer subtypes. In breast cancer, PSMB8, 9 and 10 have been found to be expressed to higher levels in hormone-positive cancers compared to other subtypes (71). Given the impact of ImP expression on cancer cells, its regulation is often altered in malignant vs healthy cells.

Notably, ImP repression is often observed in cancer. In breast cancer, retinoic acid receptor responder protein 3, a known suppressor of lung metastasis (72), has been shown to downregulate PSMB8, 9 and 10 expression through IRF1 depletion (73). In acute promyelocytic leukemia, the promyelocytic leukemia-retinoic acid receptor-α fusion protein suppresses the function of PU.1, a key transcription factor that is required for ImP expression (**Figure 2**), and all-*trans* retinoic acid, which degrades the fusion protein, was shown to restore ImP expression and is seen as a promising therapeutic option of the patients (74). Another mechanism by which cancer cells prevent ImP expression is via epigenetic silencing. For instance, in mesenchymal NSCLC, STAT3 recruits methyltransferases that hyper-methylate the *PSMB* promoters and therefore prevent ImP expression (75). Likewise, acute myeloid leukemia of the M3 subtype show heavy DNA methylation in the region of *PSMB* promoters and expresses low ImP levels (68). Finally, CD28 co-stimulation interferes with DNA methylation of the ImP promoters in myeloma models, which prevents expression (76). The aberrant expression of the ImP by cancer cells affects the disease at all steps from carcinogenesis to dissemination and also modulates treatment responses. As such, a deeper understanding of its interplay with cancer is warranted in order to harness its activity to improve outcomes.

## 6 The ImP in oncogenesis and disease progression

The ImP plays cancer-specific roles at various stages of the disease (**Table 3**). While its role in cancer immunoediting is established (34, 77, 78), it also impacts tumor onset. As such, PSMB8 is a key driver of oncogenesis in many cancers, including colitis-induced cancers, which are associated with chronic inflammatory conditions (79, 80). Also, another group has demonstrated that *PSMB8* knockout (KO) mice are resistant to chronic inflammation and fail to develop tumors upon exposure to carcinogens (55). Additionally, Leister et al. demonstrated that pro-tumorigenic factors such as cyclooxygenase-2, interleukin-6 and interleukin-1β were lower in ImP KO mice (49). Also, correlative studies predicted that PSMB8 has a carcinogenic role in lower-grade glioma, uveal melanoma and pancreatic adenocarcinoma (69). Additionally, in some subtypes of acute myeloid leukemia, genomic rearrangements involving the lysine methyltransferase 2A gene *KMT2A* yield fusion proteins that drive oncogenesis (81). Interestingly, a study found that inhibiting PSMB8 with the drug ONX-0914 inactivated *KMT2A*, therefore suggesting a pro-AML role for this ImP subunit (82). In hepatocellular carcinoma, PSMB8 was found to be co-expressed with zinc finger protein 655 a protein that supports cancer proliferation and tumour establishment (83, 84) and PSMB8 knock-down significantly reduced disease severity (85). Taken together, these studies support a pro-cancer role for the ImP in specific cancer types.

**Table 3:** Impacts of ImP expression in different cancer types.

Cancer	ImP subunit	Impact
Colitis-associated colon cancer	↑ PSMB8	Increased inflammation-driven tumorigenesis in mice (49, 55) Decreased survival (55)6/6/25 9:29:00 AM
Uveal melanoma		Predictive carcinogenic role in patients (69)
Pancreatic adenocarcinoma		Predictive carcinogenic role in patients (69)
Gastric cancer		Decreased survival (101)
Hepatocellular carcinoma	↓ PSMB8	Reduced disease severity (85)
Leiomyosarcoma	↓ PSMB9	Unfavorable prognosis in patients (87)
Glioma	↑ PSMB8	Predictive carcinogenic role in patients (69)
	↑ PSMB9	Increased signatures for CD4 and CD8 T cells, B cells, macrophages and NK cells (50) Poor prognosis in patients (52, 61) 6/6/25 9:29:00 AM
Bladder urothelial cancer	↑ PSMB8 ↑ PSMB9 ↑ PSMB10	Improved survival (52, 61)
Sarcoma		Improved survival (52, 61)
Thymic cancer		Improved survival (7)
Pancreatic cancer		Decreased survival (69)
Clear cell renal cell carcinoma		Decreased survival (7)
Acute myeloid leukemia	↓ PSMB8	Inactivation of *KMT2A* oncogenic proteins (82)
	↑ PSMB8 ↑ PSMB9 ↑ PSMB10	Decreased survival (7)
Myeloma	↑ PSMB8	Increased sensitivity to proteasome inhibitors (113)
NSCLC	↓ PSMB8 ↓ PSMB9 ↓ PSMB10	Increased epithelial-to-mesenchymal transition (75)
	↑ PSMB8 ↑ PSMB9 ↑ PSMB10	Restored MHC-I peptide repertoire upon demethylation of ImP subunit promoters (75) Improved survival (7, 39)
Breast cancer	↓ PSMB8	Possible immune evasion and metastasis (89)
	↑ PSMB8	CD8 T cell infiltration of tumors (43) Increased expression of IFN-stimulated genes (43)
	↑ PSMB8 ↑ PSMB9 ↑ PSMB10	Improved survival (52, 61, 63, 96)
Melanoma	↓ PSMB8	Decreased infiltrating Th1 cells (49)
	↓ PSMB8 ↓ PSMB9 ↓ PSMB10	Faster growth of B16F10 tumors in ImP-KO mice vs wild-type mice (49) Decreased effector T cells in the tumor microenvironment (49)
	↑ PSMB8 ↑ PSMB9 ↑ PSMB10	Increased presentation of MHC-I peptides derived from tumor-associated antigens (42) Improved survival (42)

Arrow pointing up means high expression. Arrow down means low expression.

Interestingly, the opposite was found for other indications. For instance, 36% of *PSMB9* KO mice were found to spontaneously develop uterine leiomyosarcoma (86). For leiomyosarcoma, data from three different cohorts demonstrated that PSMB9^low^ patients presented an increase in pathways driven by the *Src* proto-oncogene and a less favorable prognosis (87). Importantly, ImP expression also affects disease establishment and dissemination. In NSCLC, deficiencies in ImP subunits drive epithelial-to-mesenchymal transition (75), a phenomenon that contributes to tumor aggressiveness through metastasis (88). In breast cancer patient samples, PSMB8 was shown to be decreased in brain metastases, suggesting that its loss could play a role in metastasis (89). Altogether, these findings highlight the diverse roles of the ImP in cancer establishment and progression.

## 7 ImP and anti-tumor immunity

Many reports describe the ImP influencing anti-tumor immunity (90–93). For instance, subcutaneous B16F10 melanoma tumors grow faster in ImP KO mice compared to their wild-type counterparts, a phenotype that was associated with decreased effector T cells within the TME and draining lymph nodes (49). In human breast cancer, high PSMB8 expression was associated with increased intra-tumoral CD8 T cell infiltration (43). The authors also found that PSMB8 expression correlated with increased expression of the IFN-stimulated genes myxovirus resistance gene A and protein kinase R, high mobility group nucleosome binding domain 1 and high mobility group box 1 danger-associated molecular patterns, therefore indicating a pro-immune state in the presence of PSMB8 (43). Finally, PSMB8 KO mice exhibit depleted Th1 CD4 T cells within melanoma tumors, which was shown to allow for faster progression (49). Given that T cells are important players of anti-tumor immunity, these effects of the ImP are likely to impact their capacity to recognize and eliminate cancer cells. To do so, T cells scan the antigenic peptides that are presented at the cell surface and specifically unleash their cytotoxic activity against the cells that are not recognized as self.

The collection of peptides that are presented at the cell surface by MHC molecules constitutes the peptide repertoire (or immunopeptidome) (94). Interestingly, dendritic cells from PSMB8/9/10 KO mice have been shown to be defective in the presentation of multiple epitopes (95). As such, the contribution of the ImP to the immunopeptidome in the context of cancer has emerged as a new avenue to increase anti-tumor immunity. Counter-intuitively, many studies reported that the ImP limited the presentation of some tumor epitopes by dendritic cells and B cells (96–98). These results, together with the established differences in the peptides yielded from protein degradation by the CP vs ImP further support an impact of the ImP on the peptide repertoire.

The peptide repertoire of cancer cells is important for their elimination by T cells, and is also a key factor in the efficacy of cancer vaccines. For a peptide vaccine targeting the cancer driver epidermal growth factor receptor variant III (EGFRvIII), a mutation in the peptide sequence enhanced ImP processing and translated into better therapeutic efficacy in the GL261-EGFRvIII glioblastoma model (99). Also, human cell lines of NSCLC were reported to have depleted repertoires of MHC-I peptides, which could be rescued by treatment with the DNA methyltransferase inhibitor 5-aza-2’-deoxycytidine (which demethylates ImP subunit promoters) or IFNγ, both of which increase Imp expression. Further peptide repertoire analyses and *in vitro* cell-mediated cytotoxicity assays confirmed the therapeutic potential of the approach (75). Altogether, these studies highlight the contribution of the ImP in shaping anti-tumor immune responses through peptide processing and presentation. As such, its expression affects cancer outcomes.

## 8 ImP and cancer outcomes

Given the ability of the ImP to enrich the tumor milieu with multiple key players of anti-tumor immunity, many groups have studied the link between ImP expression and the prognosis of cancer patients. In breast cancer, NSCLC, bladder urothelial cancer, sarcoma, mesothelioma, melanoma and thymic cancer, elevated levels of PSMB8, 9 or 10 mRNA correlate with improved survival (7, 39, 43, 45, 49, 69, 75). This has been very well studied in breast cancer for which *PSMB8* expression was linked to better disease-free survival in patients presenting with lymph node metastases at the time of diagnosis (43). Furthermore, we previously published that triple-negative breast cancers had better prognoses when PSMB8 and 9 protein expression were detected in cancer cells (100). For some other cancers, ImP is associated with a worst prognosis. For example, PSMB8 expression decreased survival in gastric cancer (101) and PSMB9 expression correlated with poor outcomes in glioma (45). Co-expression of the three subunits was also linked to poor outcomes in pancreatic cancer, clear cell renal cell carcinoma and AML (7). These studies highlight the context-dependent effects of ImP expression in cancer.

From a therapeutic standpoint, ImP expression has also been reported to affect treatment responses, notably in the context of immunotherapies. In the last decade, immune checkpoint blockade has revolutionized cancer treatment (102, 103), and many studies have now shown that ImP expression is predictive of treatment response to immune checkpoint inhibitors in melanoma, NSCLC, breast, bladder and thymus cancer (7, 42, 58). Interestingly, ImP expression was found to be a superior at predicting treatment responses compared to the tumor mutational burden (42) and the expression of other IFNγ-induced gene (58). More work is still required to understand the molecular mechanisms linking ImP expression and immune checkpoint blockade sensitivity. The many impacts of ImP expression on the prognosis of cancer patients are summarized in **Table 3**.

For most hematological cancers, the impact of ImP expression remains poorly characterized (104). Interestingly, the ImP governs protein homeostasis and supports the survival of these cancers, notably because it constitutes the majority of the proteasome pool (105). This ImP dependency makes hematological malignancies ideal candidates for ImP inhibition as a treatment (104, 106–111). Accordingly, the ImP is gaining interest in the field of cancer therapy and its inhibition is being explored as a treatment for some cancers.

## 9 ImP inhibition in cancer

Inhibiting ImP activity is seen as a promising therapeutic avenue for many cancers (112, 113). The ImP inhibitors that are currently used in clinical and pre-clinical studies are summarized in **Table 4**. Proteasome inhibitors that target both the CP and the ImP were first explored and bortezomib, carfilzomib and ixazomib are currently approved by the Food and Drug Administration for treatment-refractory or relapsing multiple myeloma (112, 114, 115). These drugs target proteasome subunits with chymotrypsin-like activity (116–118) and worldwide, over 140 clinical trials are currently exploring the efficacy of these drugs against myelomas, lymphomas and leukemias. Notably, bortezomib is also approved in mantle cell lymphoma and is currently undergoing trials for other cancers although severe toxicities and the development of resistance have been reported (119).

**Table 4:** Overview of current ImP inhibitor usage in clinical and pre-clinical studies.

ImP inhibitor	Category	Cancer type	Active clinical trials in cancer*
Bortezomib	Non-selective	Myeloma	59
		Central nervous system tumors	1
		Plasmacytoma	1
		Acute lymphoblastic leukemia	3
		Acute myeloid leukemia	3
		Lymphoma	5
		Urethral cancer	1
Carfilzomib	Non-selective	Myeloma	49
		Lymphoma	1
Ixazomib	Non-selective	Myeloma	21
		Acute lymphoblastic leukemia	1
		Kidney cancer	1
		Lymphoma	2
		Urothelial carcinoma	1
ONX-0914	Selective for PSMB8	Glioblastoma Acute lymphoblastic leukemia NSCLC Colorectal cancer Gastric cancer Prostate cancer	Pre-clinical studies only
PR924	Selective for PSMB8	Myeloma Plasmacytoma Leukemias	Pre-clinical studies only
M3258	Selective for PSMB8	Myeloma	Pre-clinical studies only
IPSI-001	Selective for PSMB8	In development	Pre-clinical studies only
KZR504	Selective for PSMB9 Selective for PSMB9 Selective for PSMB9		
LU-001i			
LU-002i			
KZR616	Selective for PSMB8 and PSMB10		

*As collected from clinicaltrials.gov on May 27, 2025, with search filter “Active, not recruiting” applied for each non-selective proteasome inhibitor.

As for inhibitors that specifically inhibit the ImP, few have been developed in the last decade. As such, ONX-0914, the most studied ImP inhibitor, blocks PSMB8 and has been reported to induce cell death in pre-clinical models of glioblastoma, acute lymphoblastic leukemia, NSCLC, colorectal, gastric and castration-resistant prostate cancers (55, 107, 120–125). PR-924 is another PSMB8 inhibitor with enhanced ImP selectivity and potency compared to ONX-0914 (126). It was shown to trigger apoptosis in multiple myeloma, prolonged survival in a plasmacytoma xenograft model and was also cytotoxic against bortezomib-resistant leukemia cell lines (127, 128). Another compound targeting PSMB8 is M3258, which was shown to efficiently induce the apoptosis of tumor cells in multiple myeloma xenograft models and presenting a favorable safety profile (110, 129). Additional ImP inhibitors have recently been developed, including IPSI-001, KZR504 and LU-001i, which all target PSMB9, as well as LU-002i which targets PSMB10 (130–133). The small molecule KZR616, which inhibits both PSMB8 and 10 (134), is the only selective ImP inhibitor to currently be tested clinically, although not for a cancer indication, but as a treatment for autoimmunity (NCT04628936, NCT04039477, NCT03393013, NCT04033926, NCT05569759, NCT05781750). The various clinical trials testing proteasome inhibitors against cancer are summarized in **Table 4**. As we deepen our understanding of the ImP in the context of cancer, it is likely that its therapeutic potential will be established, leading to additional clinical studies aiming at using ImP-selective inhibitors against cancer.

## 10 Concluding remarks

**Figure 3:**
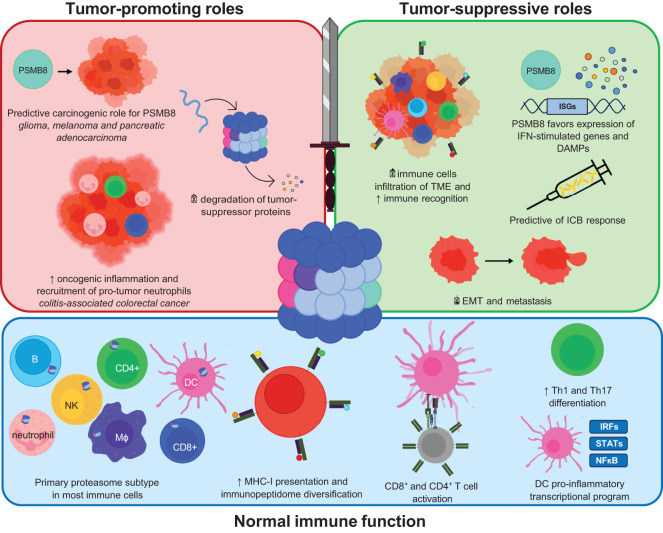
Schematic representation of the functional outcomes of ImP activity in normal immune function, tumor-promotion and tumor-suppression. The roles of the ImP in normal immune functions includes increased MHC-I presentation, immunopeptidome diversification, T cell activation, increased differentiation of CD4+ T cells into Th1 or Th17 cells or influencing the transcriptional program of dendritic cells. Amongst its tumor-promoting roles, the ImP has a predictive carcinogenic role in some cancers and degrades various tumor-suppressor proteins. The ImP also drives oncogenic inflammation in colitis-associated cancers. It may also increase immune cell infiltration in the TME and trigger danger-associated molecular patterns (DAMPs).

While the ImP was discovered almost 30 years ago, its role in cancer was only revealed in the last decade. We are currently expanding our knowledge of its dichotomous roles at the different stages of cancer progression, which will likely affect future therapies (**Figure 3**). Importantly, while most cancers exhibit increased ImP expression compared to healthy tissues, and many ImP inhibitors show promising results in pre-clinical models of the disease, the ImP also supports anti-tumor immunity, which is important for tumor control. This tug-of-war between dampening harmful ImP activity and preserving its pro-immune functions remains to be fully understood. Future research will likely aim at understanding this balance, uncovering optimal ImP targeting strategies based on cancer type, harnessing the potential to mount a favorable anti-tumor immune response, and combining Imp modulation with existing therapies.
